# Supplementary material for: Pre-neuronal processing of haptic sensory cues via dispersive high-frequency vibrational modes
Source: Sci Rep. 2023 Sep 1;13:14370. doi: 10.1038/s41598-023-40675-8 (PMC10474056; doi:10.1038/s41598-023-40675-8)
Supplement: Supplementary file 1 — Supplementary Information 1. [file 41598_2023_40675_MOESM1_ESM.pdf]

SUPPLEMENTARY INFORMATION

PRE-NEURONAL PROCESSING OF HAPTIC SENSORY CUES VIA DISPERSIVE HIGH-FREQUENCY VIBRATIONAL MODES

Yu Ding and Yurii Vlasov

Supplementary Movies:

SI Movie 1: Spectral analysis of whisker interaction with a pole.

Voltage trace (top) of a C1 21.5mm mouse whisker swept over a pole at 10mm distance from the tip. Video (bottom) is composed of 10 CMW spectrograms calculated from 10 consecutive sweeps. Note highly reproducible regular pattern of modal vibrations at times corresponding to a whisker slipping off the pole (downward red arrows) and collision with the pole (upward arrows). Note also, that at the first touch the eigenmodes pattern corresponds to a shorter whisker arc than for a slip-off. Corresponding upward chirp in eigenmodes frequencies is schematically shown by white dashed lines.

SI Movie 2: Spectral analysis of whisker interaction with a textured surface.

Voltage trace (top) of a B1 40mm rat whisker swept over a sandpaper with p60 grit value located at 2mm distance from the tip. Video (bottom) is composed of 10 CMW spectrograms calculated from 10 consecutive CF sweeps. Note highly reproducible regular pattern of modal vibrations at times corresponding to a whisker slipping off the sandpaper surface (downward red arrow) and first contact with the sandpaper (upward arrow). Events *a*, and *b* discussed in relation to Fig.4A,B are marked by downward red arrows.

SI Movie 3: Whisker swipe over grating – Fig.S6.

Background-subtracted 1000fps video of a mouse whisker scanned over a 2mm pitch grating (same results as in Fig.S6). Note blurring of the images when whisker is vibrating at frequencies beyond 1000Hz.

SI Movie 4: High-speed collisions during sweeping over a grating – Fig.5.

Background-subtracted video of a C2 mouse 24.1mm long whisker during a single CB sweep over a grating recorded at 1460fps (same results as in Fig.5). The image of the whisker (thinner bottom trace) is accompanied by an image of the whisker shadow projected on a screen (thick upper trace). Note, that the view of a whisker tip is blocked by grating teeth. The position of the teeth at the plane of a sweep (left) and the microphone base (right) are shown by rectangular shapes. Events marked 1, 2, and 3 correspond to interaction with individual grating teeth as in Fig.5. Overlaid are the whisker shapes extracted from consecutive 160 frames.

SI Movie 5: Spectral analysis of interaction of a whisker with a grating – Fig.5.

Typical voltage trace (top) of a mouse C2 whisker with 24.1mm arc during a single CB sweep over a grating. Events marked 1, 2, and 3 correspond to interaction with individual grating teeth as in Fig.5. Video (bottom) is composed of 10 CMW spectrograms calculated from 10 consecutive CB sweeps. Time for each spectrogram is aligned to the start of event 2.

Supplementary Figure S1.

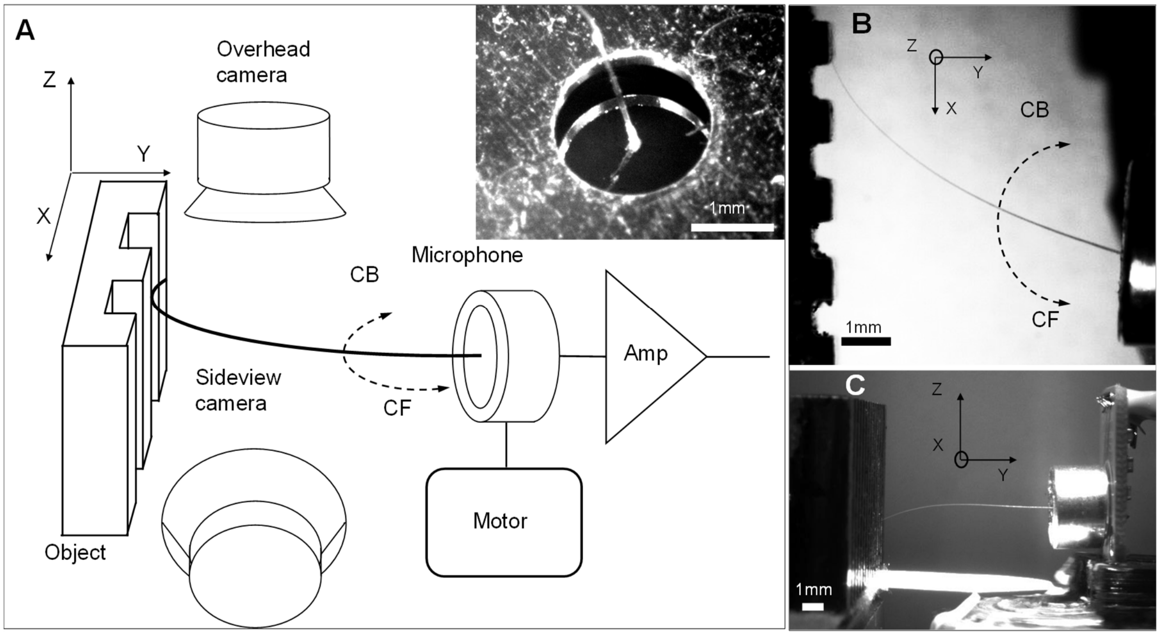

Fig.S1. Whisker micromotions acquisition system.

(**A**) Schematic of the experimental setup. Inset: Photograph of a whisker glued to the electret membrane of electret condenser microphone. Vibrations induced on the membrane were transformed into voltage traces, amplified, and recorded. The microphone was swept in the *xy* plane using a stepper motor. Whisker intrinsic curvature was oriented to align with the sweeping plane thus defining convex forward (CF) and convex backward (CB) directions. An object (here a grating) was placed at *y* distance from a whisker base, deflecting the whisker during sweeping motion. **Inset:** microphotograph of a whicker follicle glued to microphone base.
(B) A frame image of whisker micromotions in the *xy* plane captured with the overhead camera.
(C) A frame image in the *yx* plane captured with the side view camera.

Supplementary Fig.S2

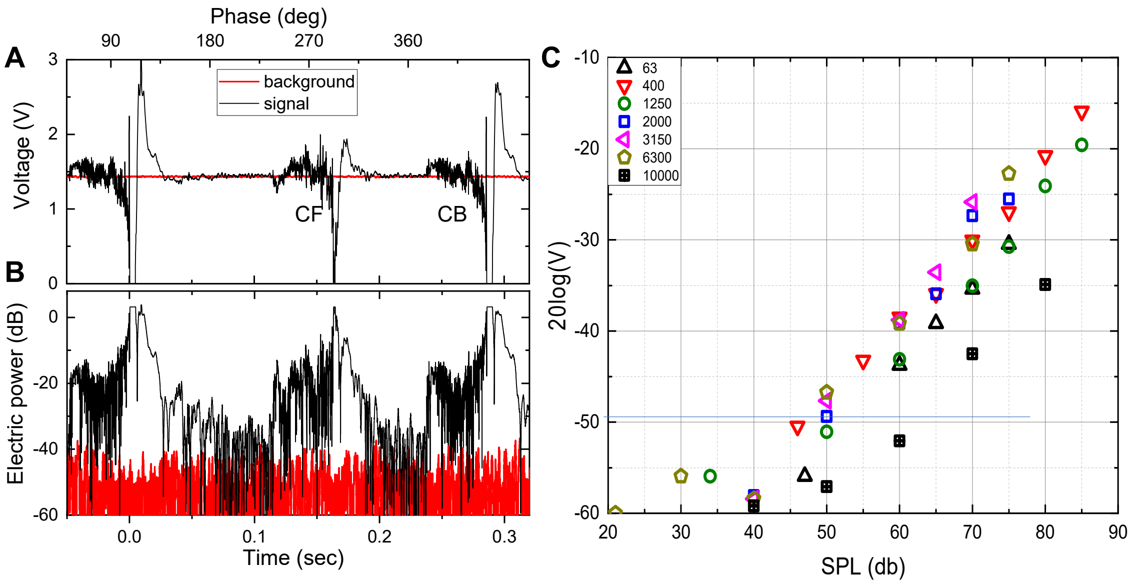

Fig.S2. Calibration of acoustic system.

(A) Voltage trace recorded with a C1 mouse whisker (black) with a total arc length of 22mm swept at 3.5Hz rate against the pole located 2mm apart from the whisker tip. For comparison, a background trace (red) without motor movement is recorded.

(B) Electric power calculated from voltage traces for signal (black) and background (red) demonstrating over 50dB dynamic range.

(C) The electric power (dB) from the electret microphone with attached whisker is plotted as a function of the acoustic field strength (SPL dB). Different symbols with different colors represent calibration at different frequencies (63-10,000Hz).

Supplementary Fig. S3.

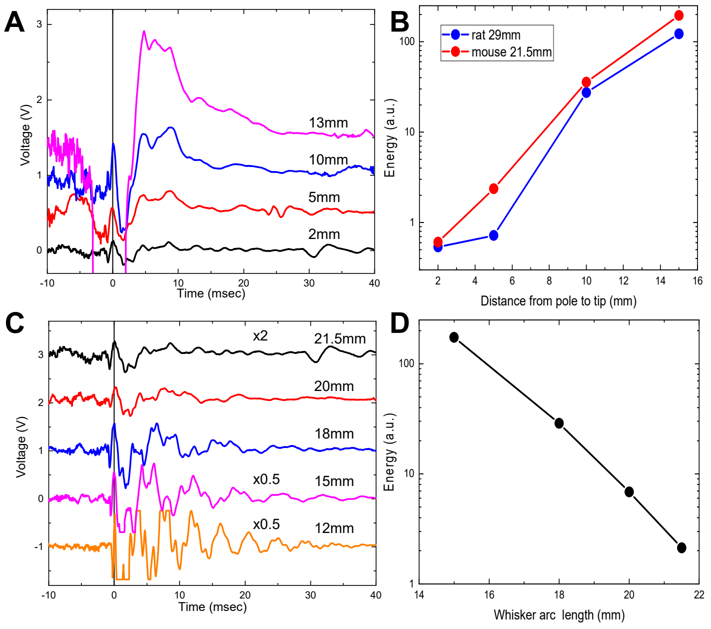

Fig.S3. Interaction with a pole.

(A) Voltage traces recorded with a C1 mouse whisker with a total arc length of 21.5mm swept at 3.5Hz rate against the pole located at different distances from the whisker tip. Traces are shifted vertically by 0.5V with respect to each other for clarity.
(B) Energy of vibrations calculated as an integral under the squared voltage traces in A) for [0..30] ms time interval.
(C) Voltage traces recorded with a C1 mouse whisker that is consecutively trimmed form initial 21.5mm to 12mm total arc length. The distance between a pole and the whisker tip is kept constant as 2mm. Traces are shifter vertically by 1V with respect to each other for clarity. Voltage traces for 12, 15, and 21.5mm are scaled with the multipliers shown. Note amplitude clipping of 12mm voltage trace due to saturation of an op-amp amplifier.
(D) Energy of vibrations calculated as an integral under the squared voltage traces in C) for [0..30] ms time interval.

Supplementary Fig. S4.

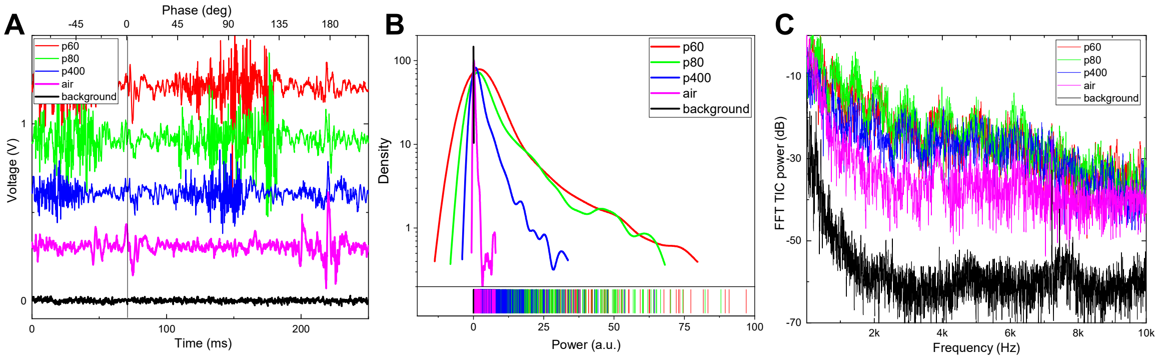

Fig.S4. Interaction with a surface.

(A) Voltage traces recorded with a C1 mouse whisker trimmed to a total arc length of 21.5mm swept at 3.5Hz rate against the sandpapers with different grit numbers. The sandpapers are located at 2mm from the whisker tip. Traces are shifted vertically by 0.25V with respect to each other for clarity. Traces for scanning of a whisker in the air (magenta) and the background noise level with scanning stopped (black) are shown for comparison.

(B) Distribution and rug plots for traces in A).

(C) FFT power spectra for voltage traces in A).

Supplementary Fig. S5.

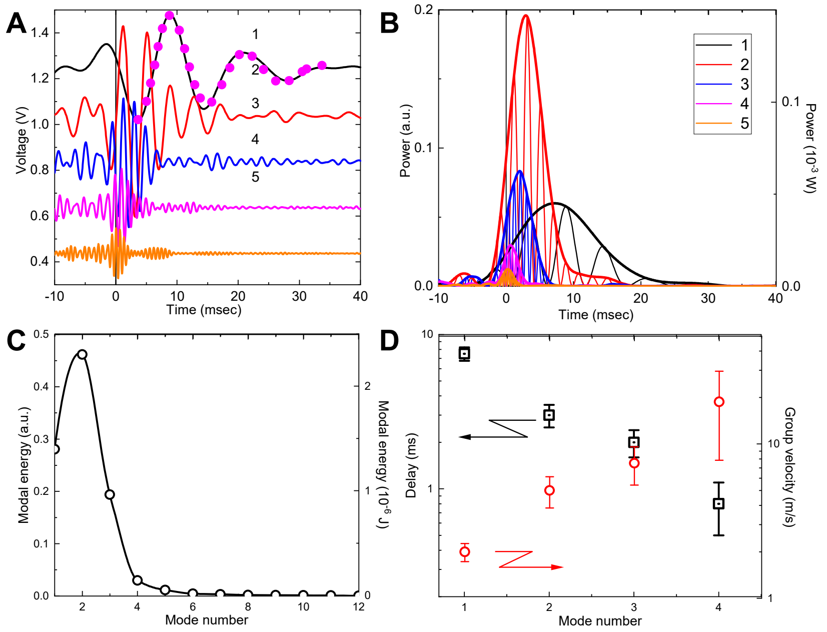

Fig.S5. FFT bandpass filtering procedure.

(A) Voltage traces recorded with a C1 mouse whisker (black) with a total arc length of 20mm swept at 3.5Hz rate against the pole located 2mm apart from the whisker tip. Traces marked 1 through 5 correspond to FFT-filtered bands according to filters cutoffs in the SI Table S2. Filtered spectra are shifted vertically by 0.2V for clarity. The dotted magenta curve on top of spectrum 2 is a fitting using a damped sinusoidal function.

(B) Plot of squared voltages in A) and their envelopes. Modal power is proportional to the magnitude of the envelope.

(C) Modal energy calculated as an integral under the envelope over a [-5..30] ms interval. Note that second order mode has the highest power.

(D) Modal group delay and group velocity calculated at the maxima of the power envelopes for corresponding modes in B).

Supplementary Fig. S6.

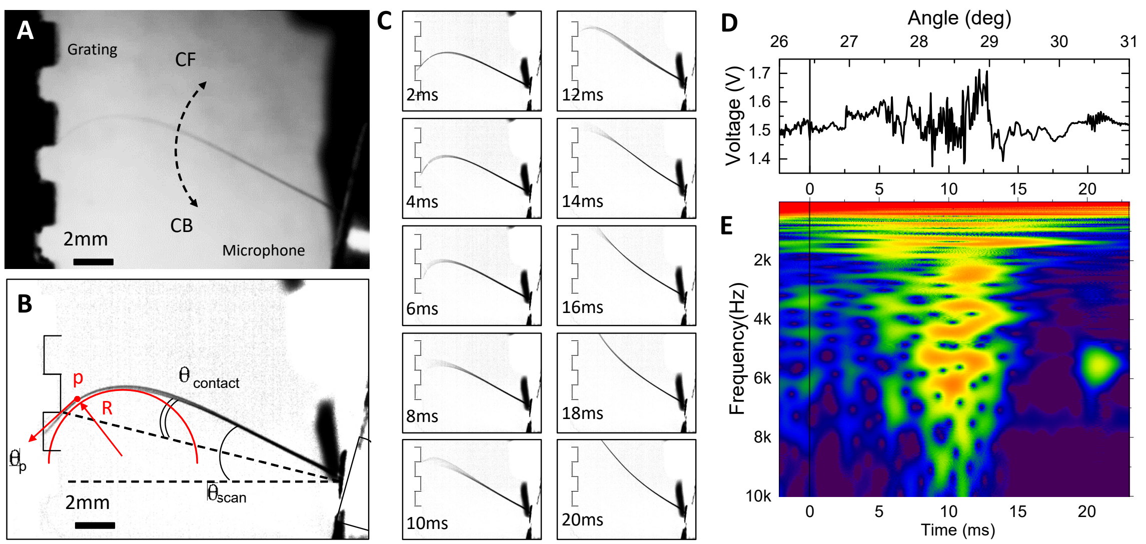

Fig.S6. Video postprocessing.

(A) Single frame of a video (SI Movie.3) recorded at 1000fps of a mouse whisker swiped over a grating.
(B) Background subtracted frame with schematics for determination of the contact angle, angle at the tip (at point p) and curvature determination.
(C) Series of background subtracted frames that are time-synchronized with the microphone voltage recording. Every second frame of a 1000fps video (SI Movie.3) is shown.
(D) Voltage trace that is time synchronized to a video in C).
(E) CMW spectrogram for D).

Supplementary Table S1. Measured parameters of whiskers

#	Whisker	R_b (μm)	S_{tot} (mm)	$Slope_R$ (x10^{-3})	Bending (A)
1	Rat B1	60	40.0	1.45	1.0
2	Mouse C1	37	21.4	1.53	1.9
3	Mouse C2	35	24.1	1.48	1.1

Supplementary Table S2. Example parameters for the FFT bandpass filtering.

**FFT band number**	**Mode central frequency (Hz)**	**Lower cutoff (Hz)**	**Upper cutoff (Hz)**
1	90	10	150
2	270	150	400
3	550	400	700
4	1000	700	1200
5	1600	1200	1800
6	2300	1900	2600
7	3300	2600	3700

Supplementary Table S3: Dimensionless eigenmode coefficients for truncated conical beam

	0	0.1	0.2	0.3	0.4	0.5	0.6	0.7	0.8	0.9
1	8.72	8.89	9.68	11.24	13.91	18.50	26.99	45.19	96.38	367.37
2	21.15	23.06	28.73	38.04	52.96	78.19	125.31	228.39	526.42	2155.03
3	38.45	45.83	62.24	87.37	127.05	194.32	320.84	600.17	1415.76	5918.86
4	60.68	78.40	111.32	160.25	237.07	367.25	612.53	1155.28	2744.07	11539.78
5	87.83	121.19	176.29	257.04	383.43	597.56	1001.23	1895.30	4515.27	19035.68
6	119.92	174.36	257.29	377.85	566.24	885.31	1486.99	2820.22	6729.15	28405.39
7	156.94	237.98	354.37	522.74	785.52	1230.55	2069.85	3930.07	9385.75	39648.10
8	198.90	312.08	467.55	691.71	1041.31	1633.28	2749.81	5224.86	12485.10	52766.52
9	245.80	396.70	596.85	884.78	1333.61	2093.53	3526.88	6704.60	16027.19	67757.95
10	297.64	491.83	742.27	1101.96	1662.43	2611.28	4401.07	8369.29	20012.03	84623.30
11	354.43	597.49	903.83	1343.25	2027.76	3186.56	5372.39	10218.95	24439.62	103362.57
12	416.18	713.69	1081.53	1608.66	2429.63	3819.35	6440.83	12253.56	29309.97	
13	482.90	840.43	1275.37	1898.19	2868.01	4509.66	7606.40	14473.13	34623.07	
14	554.59	977.71	1485.35	2211.83	3342.92	5257.49	8869.09	16877.66	40378.92	
15	631.27	1125.54	1711.47	2549.60	3854.36	6062.85	10228.91	19467.16	46577.53	
16	712.96	1283.92	1953.74	2911.49	4402.33	6925.73	11685.86	22241.61	53218.90	
17	799.70	1452.84	2212.16	3297.50	4986.82	7846.13	13239.93	25201.03	60303.02	
18	891.40	1632.31	2486.72	3707.64	5607.85	8824.05	14891.14	28345.40	67829.90	
19	988.19	1822.33	2777.43	4141.89	6265.40	9859.50	16639.47	31674.74	75799.53	
20	1090.05	2022.91	3084.29	4600.27	6959.48	10952.47	18484.93	35189.05	84211.93	
